# Plasma Proteomic Signatures in Alkaptonuria

**DOI:** 10.3390/biology15151271

**Published:** 2026-08-03

**Authors:** Rebecca Finetti, Anna Visibelli, Bianca Roncaglia, Alfonso Trezza, Luana Peruzzi, Barbara Marzocchi, Ottavia Spiga, Mattia Cicogni, Laura Salvini, Laura Tinti, Vittoria Cicaloni, Sara Cheleschi, Marco Rossi, Cristina Tinti, Annalisa Santucci

**Affiliations:** 1 Department of Biotechnology, Chemistry and Pharmacy, University of Siena, 53100 Siena, Italy; rebecca.finetti@student.unisi.it (R.F.); bianca.roncaglia@unisi.it (B.R.); alfonso.trezza2@unisi.it (A.T.); marzocchi@unisi.it (B.M.); ottavia.spiga@unisi.it (O.S.); annalisa.santucci@unisi.it (A.S.); 2 UOC Medicina Molecolare e Genetica, Azienda Ospedaliera Senese, 53100 Siena, Italy; 3 UOC Laboratorio Patologia Clinica, Ospedale S. Maria alle Scotte, AOU Senese, 53100 Siena, Italy; 4 Centro della Scienza e della Tecnica, Polo Universitario Grossetano, Via Ginori 41, 58100 Grosseto, Italy; 5 Fondazione Toscana Life Sciences, Strada del Petriccio e Belriguardo, 53100 Siena, Italy; m.cicogni@toscanalifesciences.org (M.C.); l.salvini@toscanalifesciences.org (L.S.); l.tinti@toscanalifesciences.org (L.T.); v.cicaloni@toscanalifesciences.org (V.C.); s.cheleschi@toscanalifesciences.org (S.C.); m.rossi@toscanalifesciences.org (M.R.); c.tinti@toscanalifesciences.org (C.T.); 6 Industry 4.0 Competence Center ARTES 4.0 Viale Rinaldo Piaggio, 56025 Pontedera, Italy; 7 MetabERN, Department of Biotechnology, Chemistry and Pharmacy, University of Siena, Via Aldo Moro, 53100 Siena, Italy

**Keywords:** Alkaptonuria, rare diseases, proteomics, inflammation and complement system, functional enrichment

## Abstract

Alkaptonuria (AKU) is a rare metabolic disorder caused by homogentisic acid accumulation and characterised by ochronosis, oxidative stress, chronic inflammation, and progressive connective tissue damage. This study aimed to define the circulating proteomic alterations associated with AKU and assess their relationship with nitisinone treatment. Plasma samples from 11 patients with AKU and 6 age- and sex-matched healthy controls were analysed by liquid chromatography coupled to tandem mass spectrometry using label-free quantification. Differentially abundant proteins were identified using thresholds of |log_2_FC| ≥ 1 and Benjamini–Hochberg false discovery rate ≤ 0.01, followed by functional enrichment and treatment-stratified analyses. Twenty-two proteins were differentially abundant between AKU patients and controls. Complement components (C1R, C1S, C9, C4BPA, CPN2), fibronectin, clusterin, PGLYRP2, and haemoglobin subunits showed increased abundance, whereas most immunoglobulin chains, kallikrein, apolipoprotein A2, and alpha-1-antitrypsin showed decreased abundance. Functional enrichment highlighted complement activation, B-cell-mediated and humoral immune responses, immunoglobulin-related functions, platelet activation, and erythrocyte gas-exchange pathways. Correlation analysis linked several proteins, particularly CPN2, APOA2, C1R and C1S, to core biochemical parameters of disease activity. Treatment-stratified analysis identified fourteen proteins that remained significantly altered in both treated and untreated patients, forming a treatment-resistant core of the signature, while several complement-, coagulation-, and lipid-related proteins were significant only in one treatment subgroup. These findings define an AKU plasma proteomic signature dominated by complement activation and humoral immune alterations, together with extracellular matrix, erythrocyte-, and coagulation-associated changes. The persistence of most alterations across treatment groups suggests that residual systemic proteomic dysregulation remains despite nitisinone treatment.

## Simple Summary

Alkaptonuria (AKU) is a rare inherited metabolic disorder caused by the accumulation of homogentisic acid, which progressively damages cartilage and other connective tissues. Although AKU is associated with chronic inflammation and oxidative stress, its effects on circulating proteins remain incompletely understood. In this study, we compared the plasma protein profiles of 11 patients with AKU and 6 age- and sex-matched healthy controls. Twenty-two proteins showed significant differences between the two groups. The main alterations involved proteins of the complement system, circulating immunoglobulins, fibronectin, clusterin, kallikrein, apolipoprotein A2, and haemoglobin-related proteins, indicating changes in immune regulation, extracellular matrix homeostasis, coagulation, and erythrocyte-associated processes. Fourteen of these alterations were also observed in both nitisinone-treated and untreated patients, suggesting that a substantial part of the circulating proteomic signature persists despite treatment. These findings improve our understanding of the systemic molecular changes associated with AKU and provide a basis for larger studies investigating circulating biomarkers of disease activity and treatment response.

## 1. Introduction

Alkaptonuria (AKU) is a rare autosomal recessive metabolic disorder caused by pathogenic variants in the *HGD* gene, which encodes homogentisate 1,2-dioxygenase, a key enzyme in tyrosine and phenylalanine catabolism [1]. Deficiency of this enzyme results in lifelong accumulation of homogentisic acid (HGA), a highly reactive metabolic intermediate that undergoes auto-oxidation and polymerization, leading to the formation of ochronotic pigment within connective tissues [2,3]. This process underlies the characteristic clinical manifestations of AKU, including early-onset spondyloarthropathy; progressive cartilage degeneration; tendon and ligament fragility; and multisystem involvement affecting the cardiovascular, renal, and musculoskeletal systems [4]. Beyond its metabolic origin, AKU is recognized as a systemic disorder characterized by chronic oxidative stress and sustained inflammatory activation [5]. Accumulated HGA acts not only as a pigment precursor but also as a potent pro-oxidant capable of modifying proteins, lipids, and extracellular matrix components. Experimental evidence from cellular, tissue, and clinical studies demonstrates that HGA induces protein carbonylation, thiol oxidation, lipid peroxidation, and cytoskeletal disruption, ultimately impairing extracellular matrix organization and tissue homeostasis [6,7]. These alterations establish a self-perpetuating pathogenic loop in which oxidative stress and inflammation reinforce each other, contributing to disease progression even before evident clinical manifestations become apparent. A major consequence of chronic inflammation in AKU is the persistent upregulation of serum amyloid A (SAA), a key acute-phase apolipoprotein synthesized predominantly by hepatocytes in response to pro-inflammatory cytokines such as interleukin-1β, interleukin-6, and tumor necrosis factor alpha [8]. Elevated circulating SAA levels have been consistently reported in AKU patients and show significant correlations with disease severity and systemic complications [9]. Importantly, AKU has been identified as a human secondary amyloidogenic disease, characterized by AA amyloid deposition in cartilage, synovium, tendon, salivary glands, adipose tissue, and cardiac valves [10]. Histological and ultrastructural analyses indicate that SAA-derived amyloid fibrils frequently colocalize with ochronotic pigment deposits, suggesting a close biochemical and spatial interplay between metabolic imbalance, inflammation, and protein aggregation. Among the SAA family members, SAA1 plays a central role in AA amyloidosis and exhibits clinically relevant genetic polymorphisms [11,12,13]. Therapeutically, nitisinone, an inhibitor of 4-hydroxyphenylpyruvate dioxygenase, is currently the only approved disease-modifying treatment for AKU [14]. This drug blocks an upstream step in tyrosine metabolism and markedly reduces HGA production and urinary excretion. While nitisinone effectively reduces metabolic stress, its impact on systemic inflammation, immune regulation, and amyloidogenic pathways remains incompletely understood, as clinical data show it fails to consistently normalize inflammatory biomarkers [15]. Despite the growing body of evidence linking AKU to systemic immune and inflammatory dysregulation, the circulating proteome of AKU patients has never been characterized in direct comparison to healthy controls. Previous proteomic investigations in AKU have focused primarily on specific biological matrices such as urine, synovial fluid, and cartilage tissue or have examined intra-cohort differences between patient subgroups without a healthy reference [5,7]. This critical gap limits the ability to distinguish disease-specific protein alterations from inter-individual biological variability and hinders the identification of robust plasma protein signatures associated with AKU pathophysiology. In this context, integrative plasma proteomics represents a powerful systems-level approach to dissect the complex molecular networks underlying AKU. Proteomic profiling of plasma can reveal coordinated changes in circulating proteins related to immunity, inflammation, extracellular matrix remodelling, and stress responses, linking metabolic dysfunction to clinical phenotype. In the present study, we performed for the first time a quantitative plasma proteomic analysis comparing a well-characterized cohort of 11 AKU patients to 6 age- and sex-matched healthy controls, using liquid chromatography coupled to tandem mass spectrometry (LC-MS/MS)-based label-free quantification (LFQ), to identify disease-associated protein signatures and their relationship with key biochemical and clinical parameters.

## 2. Materials and Methods

### 2.1. Patient Cohort and Clinical Data

Clinical and genetic data of 11 AKU patients were retrieved from the ApreciseKUre database [16]. Six healthy controls, matched to the patient cohort by age and sex, were also enrolled. For each participant, peripheral blood was collected, and plasma samples were obtained in accordance with standard procedures. Whole blood was processed to separate the plasma, which was then aliquoted and stored under controlled conditions until analysis. All plasma samples underwent quantitative proteomic analysis. Treatment status was defined based on documented adherence to nitisinone therapy at the time of blood collection. Patients and controls gave written informed consent before inclusion. The study was conducted in accordance with the approval of the Siena University Hospital Ethics Committee and in conformity with the standards set by the latest revision of the Declaration of Helsinki. To ensure patient confidentiality and compliance with ethical and data protection regulations, all samples were anonymised before analysis.

### 2.2. Sample Preparation

Plasma samples (5 µL) were diluted with 35 µL of ultrapure water, and proteins were precipitated by adding 160 µL of a 50:50 (*v*/*v*) acetonitrile/methanol solution. Samples were centrifuged for 5 min at room temperature, and the resulting protein pellet was resuspended in 50 µL of 8 M urea. Protein concentration was determined using the bicinchoninic acid (BCA) assay. An amount of 25 µg of protein was reduced with dithiothreitol (DTT; final concentration 10 mM) for 1 h at 60 °C and subsequently alkylated with iodoacetamide (IAA; final concentration 25 mM) for 45 min at room temperature in the dark. Protein digestion was performed overnight at 37 °C using trypsin at an enzyme-to-protein ratio of 1:30. The resulting peptide mixtures were dried under vacuum, desalted using solid-phase extraction (SPE), and resuspended in 20 µL of water containing 0.1% formic acid. Finally, 1 µg of peptides was injected for LC-MS/MS analysis.

### 2.3. LC-MS/MS Analysis

Peptide samples (1 µg) were analyzed by LC-MS/MS using a Q Exactive™ HF-X hybrid quadrupole-Orbitrap™ mass spectrometer (Thermo Fisher Scientific, Bremen, Germany) interfaced with an UltiMate™ 3000 RSLCnano UHPLC system (Thermo Fisher Scientific, Germering, Germany).Peptides were separated at 37 °C on an EASY-Spray PepMap™ RSLC C18 analytical column (75 µm × 150 mm, 2 µm particle size, 100 Å pore size; Thermo Fisher Scientific, Bremen, Germany) at a flow rate of 300 nL/min. Mobile phase A consisted of water containing 0.1% formic acid, whereas mobile phase B consisted of 80% acetonitrile, 20% water, and 0.1% formic acid. Peptides were eluted using gradient from 5% to 90% solvent B over 106 min. The mass spectrometer operated in data-dependent acquisition (DDA) mode. Full MS scans were acquired over an *m*/*z* range of 200–2000, and the 12 most intense precursor ions were selected for MS/MS analysis.

### 2.4. Data Processing

Protein identification and LFQ were performed using Proteome Discoverer version 2.5 (Thermo Fisher Scientific, Waltham, MA, USA) with the Sequest HT search engine against the Homo sapiens database. To ensure high-quality data, only proteins consistently detected across all three technical replicates were retained. Identification criteria required at least one unique peptide with a minimum length of seven amino acids. The analytical workflow was designed for global protein identification and label-free quantification of total protein abundance. The complete list of identified proteins, including UniProt accession numbers, protein and gene descriptions, identification confidence parameters, peptide-support metrics, and participant-level abundance values, is provided in Supplementary Table S1.

### 2.5. Statistical Analysis

Statistical analyses were performed in R (v. 4.5.1) using RStudio (v. 2025.05.1) as the integrated development environment. Differential expression analyses were conducted using the limma package (v. 3.64.3) [17], based on linear modeling and empirical Bayes moderation. Proteins were considered differentially abundant at a Benjamini–Hochberg-corrected FDR (BH-FDR) ≤ 0.01 and absolute log_2_ fold change (|log_2_FC|) of at least 1. Principal Component Analysis (PCA) was performed on the normalised, patient-averaged protein abundance matrix in R, with scaling to unit variance. Correlation analyses were carried out in Python (v. 3.11) using Pearson correlation coefficients to assess linear associations between variables.

### 2.6. Functional Enrichment Analysis

Functional enrichment analysis was performed to identify biological processes and pathways overrepresented among the differentially expressed proteins. Proteins were selected using a BH-FDR ≤ 0.01 and an |log_2_FC| ≥ 1 and were mapped to Entrez Gene IDs using the org.Hs.eg.db annotation database. Over-representation analysis was conducted in R (v. 4.5.1) via RStudio using the clusterProfiler package (v. 4.16.0) [18]. Gene Ontology (GO) [19] enrichment was performed separately for Biological Process (BP), Molecular Function (MF), and Cellular Component (CC) categories, while pathway enrichment was assessed using the Reactome database (https://reactome.org/, accessed on 15 May 2026) [20]. Enrichment significance was evaluated using a hypergeometric test with a BH-FDR cutoff of 0.01. Given the limited number of input proteins, only the top 5 enriched terms per category are reported.

## 3. Results

### 3.1. Study Cohort Characteristics

The study cohort comprised 17 participants, including 11 patients diagnosed with AKU and 6 age- and sex-matched healthy controls. The AKU group included seven males and four females, with ages ranging from 27 to 87 years, whereas the control group included four males and two females, with ages ranging from 27 to 86 years. At the time of plasma collection, seven AKU patients were receiving nitisinone treatment, while four were untreated. Plasma tyrosine, HGA, SAA levels, and chitotriosidase activity were available as part of the clinical and biochemical characterization of the AKU cohort and showed substantial inter-individual variability. These biochemical parameters were not measured in healthy controls, who were included for the proteomic comparison. Individual biochemical measurements and treatment status for each patient with AKU are reported in Supplementary Table S2. Correlation analyses involving these variables were restricted to patients with available measurements, and missing values were not imputed. The principal demographic characteristics of both groups, together with the clinical and genetic features of the AKU cohort, are summarised in Table 1.

### 3.2. Proteomic Data Preprocessing and Normalization

**Figure 1:**
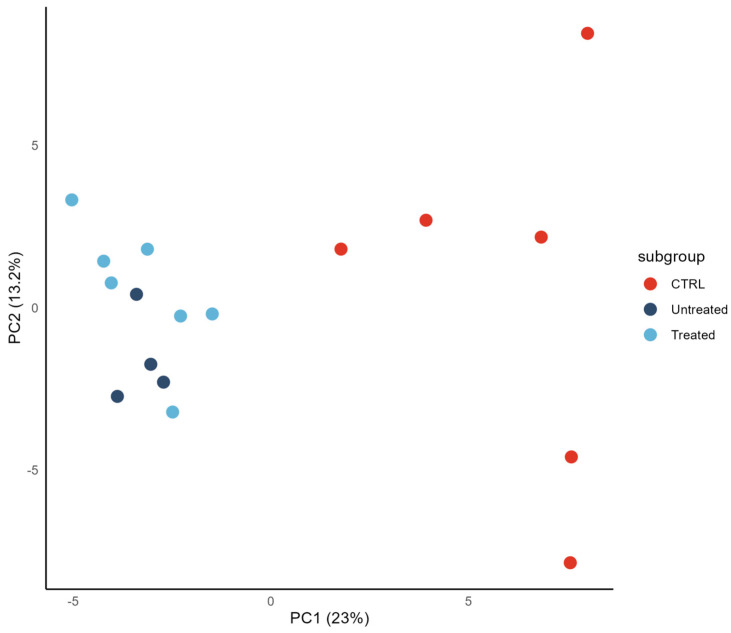
PCA of the normalised plasma protein abundance matrix. Each point represents one participant (averaged across technical replicates), coloured by clinical subgroups: healthy controls (red), untreated AKU patients (dark blue), and nitisinone-treated AKU patients (light blue). Percentages on each axis indicate the proportion of total variance explained by PC1 and PC2.

Quantitative proteomic data were generated by LC-MS/MS and processed to retain only high-confidence protein identifications. A total of 350 proteins were identified across the study cohort. Proteins with more than 25% missing values were excluded, retaining 101 proteins for downstream analysis, and the remaining missing values were imputed using a k-nearest neighbour (k-NN) approach, as reported in Supplementary Figure S1. Median normalization was then applied to minimize systematic inter-sample variability. To assess the global structure of the proteomic dataset and evaluate potential separation between the groups under study, PCA was performed on the normalised, patient-averaged abundance matrix (see Figure 1). The first two principal components accounted for 23% and 13.2% of the total variance, respectively. AKU patients were clearly separated from healthy controls along PC1, whereas treated and untreated patients largely overlapped.

### 3.3. Differential Plasma Protein Expression Between AKU Patients and Healthy Controls

**Figure 2:**
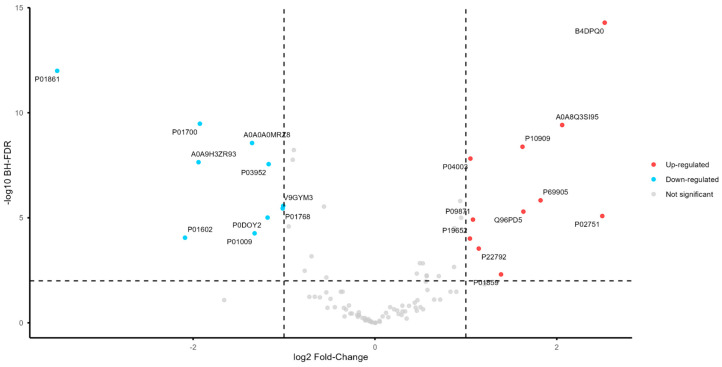
Differential plasma protein expression between AKU patients and healthy controls. Volcano plot showing the results of differential expression analysis comparing AKU patients with healthy controls. The x-axis represents the log_2_ fold change calculated as AKU/CTRL, whereas the y-axis represents −log_10_(BH-FDR). Vertical dashed lines indicate the fold-change threshold (|log_2_FC| = 1), and the horizontal dashed line indicates the significance threshold (BH-FDR = 0.01). Proteins with significantly higher abundance in AKU patients are shown in red, proteins with significantly lower abundance are shown in blue, and non-significant proteins are shown in grey. Differentially abundant proteins are labelled with their UniProt accession code.

Differential expression analysis was performed to identify plasma proteins associated with AKU by comparing the proteomic profiles of AKU patients with those of healthy controls. Using a significance threshold of |log_2_FC| ≥ 1 and BH-FDR ≤ 0.01, a distinct set of differentially abundant proteins was identified, as shown in the volcano plot in Figure 2.

Differentially abundant proteins identified between AKU patients and healthy controls are summarised in Table 2, together with their direction of change and principal biological function.

### 3.4. Functional Enrichment Analysis of AKU-Associated Differentially Abundant Proteins

**Figure 3:**
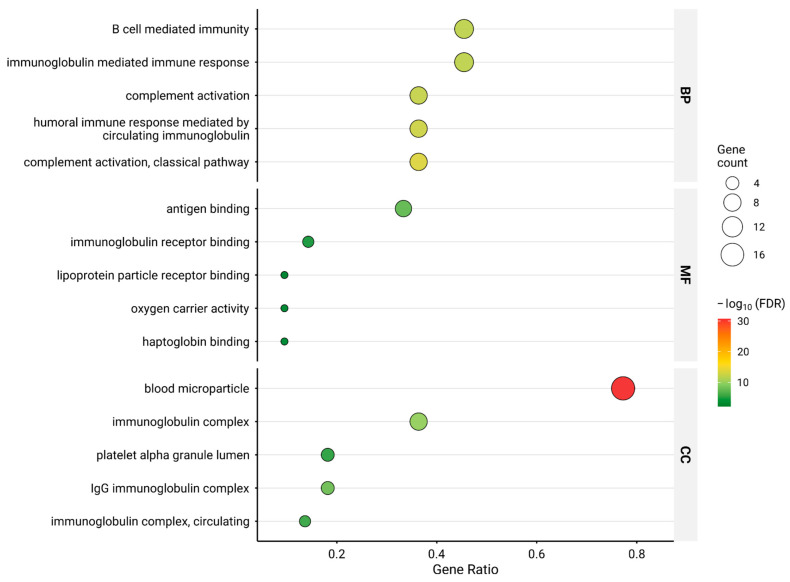
GO enrichment analysis of proteins differentially abundant between AKU patients and healthy controls. Dot plot showing the top 5 significantly enriched terms for the BP, MF, and CC categories. The x-axis represents the Gene Ratio, dot size indicates the number of proteins associated with each term, and colour represents −log10(BH-FDR).

**Figure 4:**
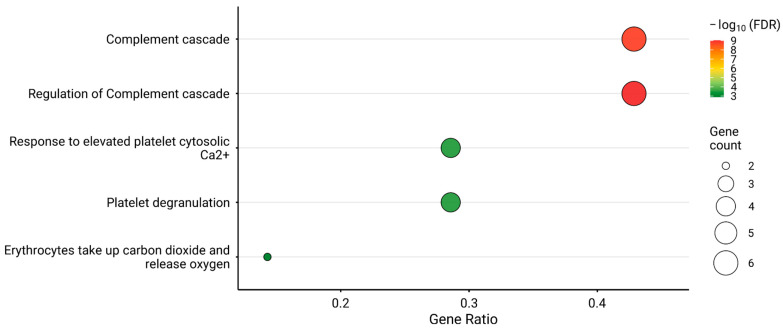
Reactome pathway enrichment analysis of proteins differentially abundant between AKU patients and healthy controls. Dot plot showing the top 5 significantly enriched Reactome pathways. The x-axis represents the Gene Ratio, dot size indicates the number of proteins associated with each pathway, and colour represents −log10(BH-FDR).

Functional enrichment analysis was performed on the 22 proteins differentially abundant between AKU patients and healthy controls (Table 2). The top 5 significantly enriched GO terms per category (BH-FDR ≤ 0.01) are shown in Figure 3, while the top 5 enriched Reactome pathways are presented in Figure 4. GO BP analysis highlighted B-cell-mediated immunity, immunoglobulin-mediated immune response, complement activation, humoral immune response mediated by circulating immunoglobulin, and activation of the classical complement pathway. At the molecular function level, the enriched terms included antigen binding, immunoglobulin receptor binding, lipoprotein particle receptor binding, oxygen carrier activity, and haptoglobin binding. GO CC analysis revealed enrichment of blood microparticles, immunoglobulin complexes (total and circulating), platelet alpha granule lumen, and IgG immunoglobulin complexes. Reactome pathway analysis confirmed prominent involvement of the complement system, including the complement cascade and its regulation, as well as pathways related to platelet activation (response to elevated platelet cytosolic Ca^2+^ and platelet degranulation) and erythrocyte gas exchange (carbon dioxide uptake and oxygen release).

### 3.5. Correlation with Clinical Data

**Figure 5:**
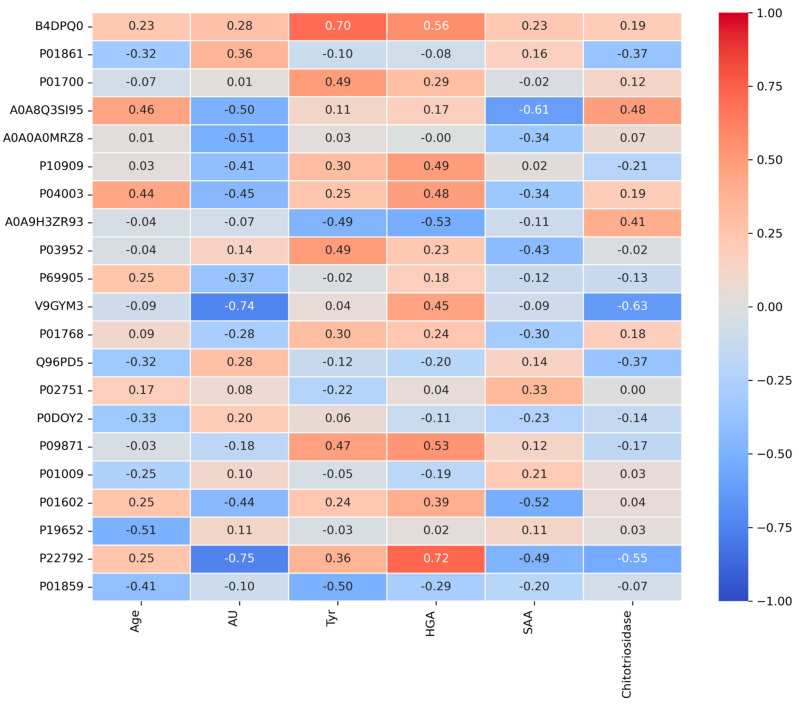
Heatmap showing Pearson correlation coefficients between differentially abundant proteins and the available clinical or biochemical parameters, calculated within the AKU cohort. Only correlations between proteins and selected clinical or biochemical variables are displayed. Colour intensity indicates the strength and direction of the correlation (red, positive; blue, negative), while numerical values represent the correlation coefficients.

Correlation analysis was performed within the AKU cohort to examine the relationship between the differentially abundant proteins listed in Table 2 and the available clinical or biochemical parameters (Figure 5). CPN2 and APOA2 showed the strongest associations overall: CPN2 correlated positively with HGA (r = 0.72) and negatively with uric acid (r = −0.75), SAA (r = −0.49) and chitotriosidase activity (r = −0.55), while APOA2 was negatively correlated with uric acid (r = −0.74) and chitotriosidase (r = −0.63) and positively correlated with HGA (r = 0.45). C1R also showed high positive correlation coefficients with both tyrosine (r = 0.70) and HGA (r = 0.56). IGLV1-47 and KLKB1 displayed a similar, weaker positive trend with tyrosine (r = 0.49), and C1S correlated positively with both HGA (r = 0.53) and tyrosine (r = 0.47), whereas IGHG3 and IGHG2 showed the opposite pattern, correlating negatively with tyrosine (r = −0.49 and r = −0.50, respectively) and, for IGHG3, with HGA (r = −0.53). C9 was negatively correlated with SAA (r = −0.61) and uric acid (r = −0.50) and positively correlated with age (r = 0.46) and chitotriosidase activity (r = 0.48). IGKV1-5 showed a comparable negative correlation with SAA (r = −0.52) and uric acid (r = −0.44), a pattern shared by IGKV3D-11 (r = −0.51 with uric acid) and C4BPA (r = −0.45 with uric acid, r = 0.48 with HGA, r = 0.44 with age). CLU correlated positively with HGA (r = 0.49), and ORM2 showed a negative correlation with age (r = −0.51). Overall, the correlation structure links the complement components C1R and C1S most directly to the core metabolic hallmarks of AKU (tyrosine and HGA), while CPN2 and APOA2 emerge as the proteins most strongly associated with disease burden and inflammatory status (uric acid, SAA, chitotriosidase). Immunoglobulin- and complement-related proteins show additional, partly divergent associations with inflammatory (SAA) and renal/metabolic (uric acid, chitotriosidase) parameters.

### 3.6. Impact of Nitisinone Treatment on Plasma Proteome

**Figure 6:**
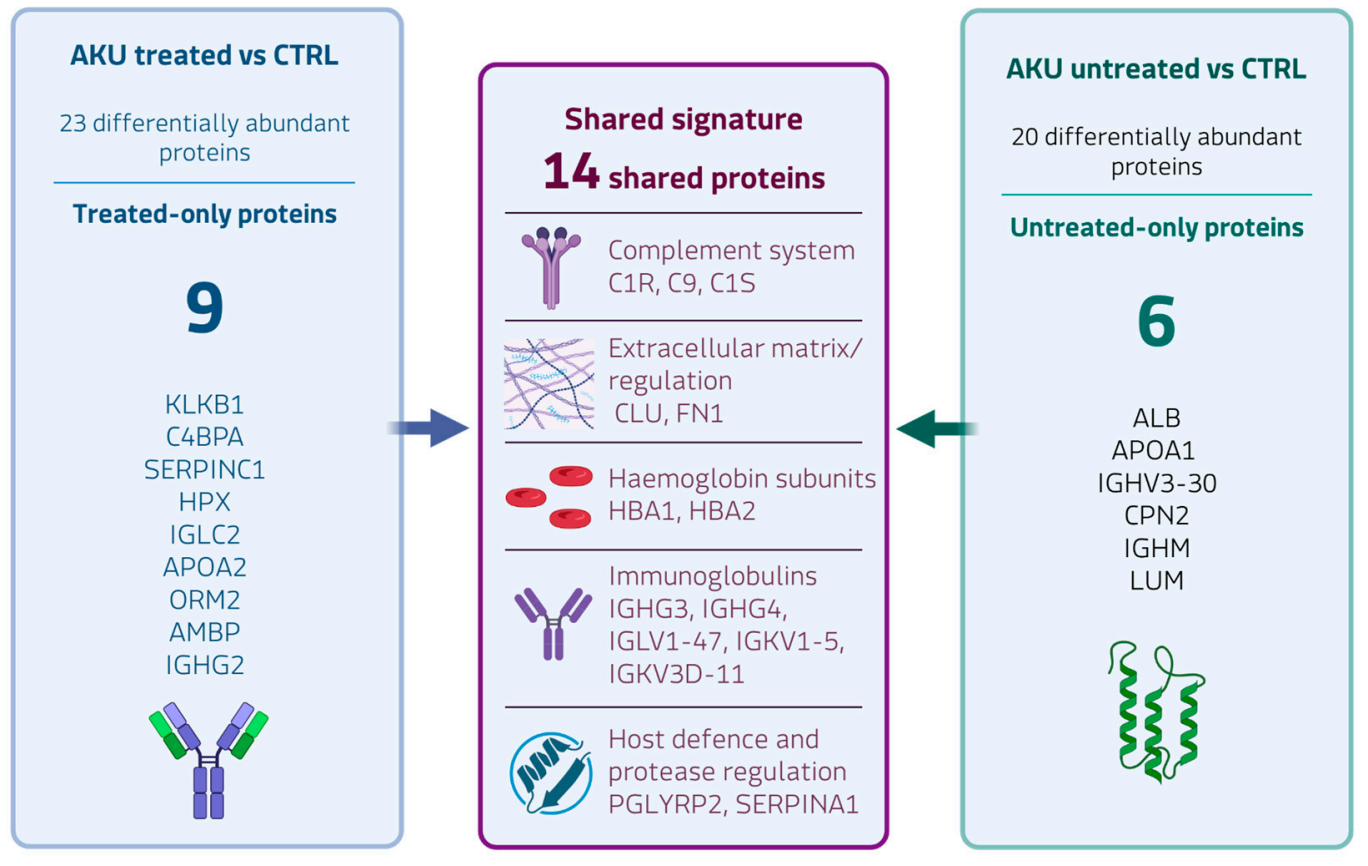
Overview of the effect of nitisinone treatment on the AKU plasma proteome: 14 proteins were shared between treated and untreated AKU patients relative to healthy controls; 9 proteins were identified exclusively in treated patients and 6 exclusively in untreated patients. Proteins were selected using |log_2_FC| ≥ 1 and BH-FDR ≤ 0.01.

To assess the influence of nitisinone treatment on the AKU-associated proteomic signature, differential expression analysis was performed separately for treated and untreated AKU patients relative to healthy controls. Using thresholds of |log_2_FC| ≥ 1 and BH-FDR ≤ 0.01, 23 proteins were found to be significantly altered in the treated patient group compared to controls (mean missing-value rate 4.3% prior to imputation), and 20 proteins were found to be significantly altered in the untreated patient group compared to controls (mean missing-value rate 2.7% prior to imputation) (Figure 6).

Fourteen proteins were found in both comparisons and showed the same direction of change. These included the complement-related proteins C1R, C1S, and C9, fibronectin (FN1), clusterin (CLU), peptidoglycan recognition protein 2 (PGLYRP2), haemoglobin subunits (HBA1; HBA2), serpin family A member 1 (SERPINA1), and immunoglobulin heavy and light chains (IGHG3, IGHG4, IGLV1-47, IGKV1-5, and IGKV3D-11). Conversely, 6 proteins were significant exclusively in the untreated-versus-control comparison (ALB, APOA1, IGHV3-30, CPN2, IGHM, and LUM), while 9 proteins were significant exclusively in treated patients versus controls (KLKB1, C4BPA, SERPINC1, HPX, IGLC2, APOA2, ORM2, AMBP, and IGHG2). Protein identities, log_2_ fold-change values, and BH-FDR values for all proteins shown in Figure 6 are reported in Table 3. Individual non-imputed LFQ intensities for these proteins are also shown in Supplementary Figure S2.

## 4. Discussion

This study provides the first plasma proteomic comparison between AKU patients and matched healthy controls using a quantitative, label-free LC-MS/MS approach with stringent statistical thresholds (BH-FDR ≤ 0.01), moving beyond the single-matrix analyses that had previously characterised the disease [5,7]. The resulting signature converged on three related axes: activation of the complement system; a broad and partly divergent remodelling of circulating immunoglobulin chains; and an extracellular matrix/erythrocyte/coagulation-associated axis involving fibronectin, haemoglobin subunits, kallikrein, and related regulatory and transport proteins.

A total of 350 proteins were identified across the cohort (Supplementary Table S1), of which 101 passed the missing-value filter used for downstream quantitative analysis. This number is considerably lower than the several thousand proteins reported by deep plasma/serum proteomic workflows performed on comparable Orbitrap instrumentation [21]. By contrast, single-shot LC-MS/MS analyses of undepleted, minimally fractionated plasma more typically identify in the range of roughly 150–400 proteins per run, closely matching the number reported here [22]. The relatively modest proteomic depth reflects the depletion-free analytical strategy rather than a limitation of the LC-MS/MS platform. Abundant plasma proteins were retained to avoid the technical variability and non-specific co-depletion associated with immunoaffinity-based depletion [23]. This approach preserved biologically informative immunoglobulin-related signals, although at the cost of reduced coverage of low-abundance proteins.

Complement components C1R and C9 were both up-regulated in AKU plasma, indicating engagement of the classical pathway and downstream membrane attack complex assembly, consistent with the established role of AKU as a secondary AA-amyloidogenic disease in which SAA-derived fibrils co-localise with ochronotic pigment in cartilage, tendon and cardiac valves [8,10]. This picture is reinforced by the concurrent up-regulation of C1S, the serine protease component of the C1 complex acting immediately upstream of C1R in classical pathway initiation, and by C4BPA, a regulatory protein that physiologically restrains classical pathway activation; its increased abundance alongside C1R, C1S and C9 suggests a coordinated, rather than unopposed, complement response, in which regulatory checkpoints are mobilised together with activating components. CPN2, the regulatory subunit of carboxypeptidase N that inactivates anaphylatoxins such as C3a and C5a, was likewise increased, further supporting a scenario of complement activation accompanied by an active counter-regulatory response rather than uncontrolled cascade progression. CLU deserves particular attention within this cascade. Beyond its classical function as a fluid-phase inhibitor of the membrane attack complex, CLU is an ATP-independent extracellular chaperone that binds misfolded and amyloidogenic proteins, limits their aggregation, and has been proposed as a translational biomarker of cartilage stress in osteoarthritis and rheumatoid arthritis [24]. Its up-regulation in AKU plasma may therefore represent a compensatory host response, attempting to buffer both complement-mediated cytolysis and HGA/amyloid-driven protein aggregation, rather than simply a marker of unopposed complement damage.

FN1 up-regulation fits the established model in which HGA-driven oxidative stress promotes extracellular matrix remodelling [6,7]. Proteolytic fibronectin fragments generated during matrix turnover are themselves recognised complement activators, engaging pathways that cross-talk with coagulation [25], raising the possibility that, in AKU, extracellular matrix damage and complement activation are not two parallel and independent processes but mutually reinforcing arms of a single pathogenic loop, testable in future work correlating fibronectin fragmentation products with complement activation fragments in longitudinal samples. This coagulation cross-talk is further echoed by the down-regulation of plasma kallikrein (KLKB1), a key protease of the contact/kallikrein-kinin system that links coagulation, fibrinolysis and inflammation and whose reduced plasma abundance may reflect altered contact-pathway activity in the context of chronic AKU-associated inflammation. Peptidoglycan recognition protein 2 (PGLYRP2), an enzyme involved in bacterial peptidoglycan degradation and innate antimicrobial defence, was also up-regulated. Its presence among the AKU-associated signature is a novel observation not previously reported in this disease and may reflect a broader innate-immune activation accompanying complement engagement.

Haemoglobin subunits (HBA1; HBA2) were likewise increased in AKU plasma, an unexpected finding given that our own earlier in vitro model of HGA-treated serum showed a decrease in haemoglobin beta subunit abundance upon direct HGA exposure [26].

The coordinated down-regulation of most immunoglobulin chains identified here (IGLV1-47, IGKV1-5, IGKV3D-11, IGHG3, IGHG4, IGHV3-30, and IGLC2) should be interpreted with caution. Immunoglobulins are structurally polymorphic proteins, and their repertoire varies substantially between individuals, independently of disease status; it has also recently been demonstrated that they are a primary cause of inter-donor plasma variability [27]. Alongside these immune-related changes, two additional proteins point to a broader systemic involvement: apolipoprotein A2 (APOA2), a structural component of high density lipoprotein, was reduced, potentially reflecting the lipid-handling perturbations increasingly recognised in chronic inflammatory states, and alpha-1-antitrypsin (SERPINA1), a key inhibitor of neutrophil elastase, was likewise decreased, consistent with ongoing proteolytic/inflammatory activity that may outpace its regulatory capacity. Conversely, orosomucoid 2 (ORM2), a classical acute-phase glycoprotein, was increased, in line with the chronic low-grade inflammatory state already documented in AKU [5].

The correlation analysis reinforced the biological coherence of these findings. CPN2 and APOA2 showed the strongest metabolic and biochemical associations within the signature. CPN2 correlated positively with HGA (r = 0.72) and negatively with uric acid (r = −0.75), SAA (r = −0.49), and chitotriosidase activity (r = −0.55), while APOA2 correlated negatively with uric acid (r = −0.74) and chitotriosidase (r = −0.63) and positively with HGA (r = 0.45), together identifying these two proteins as the strongest candidate markers of disease burden within the dataset. C1R also showed high positive correlation coefficients with both tyrosine (r = 0.70) and HGA (r = 0.56), and this pattern extended to C1S (r = 0.53 with HGA, r = 0.47 with tyrosine), directly linking classical complement pathway activation to the core biochemical hallmarks of AKU. Interestingly, IGHG3 and IGHG2 displayed the opposite pattern, correlating negatively with tyrosine (r = −0.49 and r = −0.50, respectively) and, for IGHG3, with HGA (r = −0.53), suggesting that the humoral and complement arms of the signature may respond divergently to substrate accumulation. C9 and IGKV1-5 were both negatively correlated with SAA (r = −0.61 and r = −0.52, respectively) and uric acid (r = −0.50 and r = −0.44), a pattern shared by IGKV3D-11 (r = −0.51 with uric acid) and C4BPA (r = −0.45 with uric acid), while C9 additionally correlated positively with chitotriosidase activity (r = 0.48), a validated macrophage activation marker in AKU. These associations link the identified proteomic changes to biochemical parameters currently used in clinical practice to monitor AKU severity and treatment response [15], supporting the biological plausibility of the signature as a candidate addition to existing biomarkers.

Stratification by nitisinone treatment provided further insight into the extent to which therapy influenced the observed proteomic profile. Fourteen proteins, including the complement-related proteins C1R, C1S, and C9; FN1; CLU; PGLYRP2; HBA1; HBA2; SERPINA1; and immunoglobulin chains IGHG3, IGHG4, IGLV1-47, IGKV1-5, and IGKV3D-11, remained significantly altered irrespective of treatment status, indicating a treatment-resistant core of the AKU plasma signature centred on complement activation, extracellular matrix remodelling, and humoral immune alterations. Conversely, nine proteins (KLKB1, C4BPA, SERPINC1, HPX, IGLC2, APOA2, ORM2, AMBP, and IGHG2) were significant exclusively in treated patients versus controls, while six proteins (ALB, APOA1, IGHV3-30, CPN2, IGHM, and LUM) were significant exclusively in untreated patients versus controls. The exclusivity of CPN2 to the untreated comparison is particularly notable, since this protein also emerged as one of the strongest correlates of disease burden in the whole-cohort analysis, raising the possibility that its elevation partially normalises under nitisinone. This pattern is broadly consistent with clinical data showing that nitisinone lowers HGA production but does not consistently normalise inflammatory and oxidative stress biomarkers [15]. The persistence of a complement/immunoglobulin/extracellular-matrix signature despite therapy provides a molecular rationale for exploring complement- or cytokine-targeted adjunct strategies in AKU patients who remain biochemically or clinically active despite treatment, an approach that has already achieved sustained SAA suppression and amyloid regression in AA amyloidosis of other aetiologies [28].

These findings can be compared with two earlier proteomic studies from our group, an in vitro redox-proteomic study of HGA-treated healthy serum [26] and a 2D-PAGE comparative analysis of AKU serum/plasma versus pooled healthy control [29]. CLU shows the clearest convergence, being up-regulated here, identified as a target of HGA-induced oxidation in the 2011 in vitro model, and increased in a subset of AKU samples in the 2016 gel-based study, supporting its role as a central, redox-sensitive protein across independent methods and disease contexts. Other findings diverge. The 2016 study reported increased CFAB and CO3 alongside decreased C1S, whereas the present study found both C1R and C1S up-regulated, a discrepancy, specifically for C1S, plausibly reflecting the different analytical resolution of gel-based versus LC-MS/MS quantification and the non-overlapping cohorts. The reduction in most immunoglobulin chains reported here also contrasts with the similar expression of immunoglobulin spots described in 2016, likely because peptide-level LC-MS/MS resolves isoforms and constant/variable regions that co-migrate on 2D gels. This same resolution is what allowed the present study to detect the divergent, isotype-specific behaviour of IGHG2 relative to the other IgG subclasses. Finally, the antioxidant carriers central to the 2016 oxidative-stress model, namely ceruloplasmin, haptoglobin and transferrin, did not reach significance here, possibly reflecting the stricter statistical threshold applied or differences between the present, partly treated cohort and the earlier treatment-naïve one. The reduced abundance of APOA2 is, to our knowledge, a novel observation in AKU plasma, not previously reported in either the 2011 or 2016 studies, and warrants confirmation as a potential lipid-handling correlate of disease activity. One limitation of this study is that post-translational modifications were not specifically investigated, as the analytical workflow was designed for global protein identification and label-free quantification. Future studies should therefore explore disease-relevant post-translational modifications using dedicated analytical workflows. Overall, this comparison shows that the present quantitative, FDR-controlled analysis both reinforces and refines the earlier qualitative observations from our group.

## 5. Conclusions

This study provides the first direct comparison of the plasma proteome of patients with AKU and matched healthy controls using a quantitative, label-free LC-MS/MS approach. The results identify a coordinated disease-associated signature characterised by activation of the complement system, a broad and partly divergent remodelling of humoral immune components, and an extracellular matrix/erythrocyte/coagulation-associated axis. The persistence of alterations involving C1R, C1S, C9, FN1, CLU, PGLYRP2, haemoglobin subunits (HBA1; HBA2), SERPINA1, and several immunoglobulin chains (IGHG3, IGHG4, IGLV1-47, IGKV1-5, IGKV3D-11) across treatment groups suggests that these processes represent stable features of AKU rather than secondary consequences of untreated homogentisic acid accumulation alone. Nitisinone treatment was associated with a shift in the proteins that reached significance, with several complement- and coagulation-related proteins (KLKB1, C4BPA, IGHG2) and lipid- or acute-phase-related proteins (APOA2, ORM2) emerging as significant only in treated patients, and CPN2 emerging as significant only in untreated patients, raising the possibility that its elevation partially normalises under therapy. However, treatment did not fully correct the underlying complement and humoral immune signature. Although the limited cohort size requires cautious interpretation and independent validation, these findings provide a basis for developing circulating biomarkers of disease activity and treatment response and are consistent with, while also extending, our previous gel-based and in vitro proteomic observations in AKU. They also support further investigation of complement- and cytokine-directed strategies as potential adjunctive approaches for patients with persistent inflammatory or amyloidogenic activity despite nitisinone therapy.

## Figures and Tables

**Table 1:** Demographic characteristics of the AKU patients and healthy controls included in the study, together with the principal clinical, genetic, and biochemical characteristics of the AKU cohort.

Category	AKU Patients (*n* = 11)	Healthy Controls (*n* = 6)
Sex	7 males, 4 females	4 males, 2 females
Age (years)	Range: 27–87	Range: 27–86
Nitisinone treatment status	7 treated, 4 untreated	/
Plasma tyrosine	Variable across patients, elevated values in treated individuals	/
Plasma HGA	Lower levels in treated patients	/
Plasma SAA	Wide inter-individual variability	/
Chitotriosidase activity	Variable across the cohort	/

**Table 2:** Differentially abundant plasma proteins between AKU patients and healthy controls. Direction refers to protein abundance in AKU patients relative to healthy controls.

Protein	UniProt Accession	Direction	log_2_FC	BH-FDR	Brief Functional Description
C1R	B4DPQ0	Up-regulated	2.53	5.17 × 10^−15^	Initiates the classical complement pathway
C9	A0A8Q3SI95	Up-regulated	2.06	3.84 × 10^−10^	Pore-forming component of the membrane attack complex
CLU	P10909	Up-regulated	1.62	4.16 × 10^−9^	Extracellular chaperone and complement regulator
FN1	P02751	Up-regulated	2.50	8.23 × 10^−6^	Extracellular matrix adhesion and remodelling protein
PGLYRP2	Q96PD5	Up-regulated	1.63	5.08 × 10^−6^	Enzyme involved in bacterial peptidoglycan degradation
HBA1; HBA2	P69905	Up-regulated	1.82	1.48 × 10^−6^	Haemoglobin alpha subunit involved in oxygen transport
C4BPA	P04003	Up-regulated	1.05	1.52 × 10^−8^	Regulatory protein that inhibits the classical complement pathway
C1S	P09871	Up-regulated	1.08	1.22 × 10^−5^	Serine protease component of the C1 complex in the classical complement pathway
ORM2	P19652	Up-regulated	1.04	9.75 × 10^−5^	Acute-phase plasma glycoprotein involved in immune modulation
CPN2	P22792	Up-regulated	1.14	2.92 × 10^−4^	Regulatory subunit of carboxypeptidase N, inactivates anaphylatoxins
IGHG2	P01859	Up-regulated	1.39	4.95 × 10^−3^	Constant region of the IgG2 heavy chain
IGLV1-47	P01700	Down-regulated	−1.93	3.33 × 10^−10^	Immunoglobulin lambda variable region involved in antigen binding
IGHG3	A0A9H3ZR93	Down-regulated	−1.94	2.27 × 10^−8^	Constant region of the IgG3 heavy chain
IGHG4	P01861	Down-regulated	−3.50	1.01 × 10^−12^	Constant region of the IgG4 heavy chain
IGKV1-5	P01602	Down-regulated	−2.09	8.88 × 10^−5^	Immunoglobulin kappa variable region involved in antigen binding
IGKV3D-11	A0A0A0MRZ8	Down-regulated	−1.35	2.76 × 10^−9^	Immunoglobulin kappa variable region involved in antigen binding
KLKB1	P03952	Down-regulated	−1.17	2.80 × 10^−8^	Plasma kallikrein, involved in coagulation and inflammation cascades
APOA2	V9GYM3	Down-regulated	−1.01	2.70 × 10^−6^	Structural component of high-density lipoprotein, involved in lipid transport
IGHV3-30	P01768	Down-regulated	−1.01	3.58 × 10^−6^	Immunoglobulin heavy chain variable region involved in antigen binding
IGLC2	P0DOY2	Down-regulated	−1.18	9.78 × 10^−6^	Constant region of the immunoglobulin lambda light chain
SERPINA1	P01009	Down-regulated	−1.32	5.49 × 10^−5^	Protease inhibitor (alpha-1 antitrypsin), regulates neutrophil elastase

**Table 3:** Differentially abundant plasma proteins in treated and untreated AKU patients relative to healthy controls. Category refers to whether each protein was significant in both comparisons or exclusively in one.

Protein	UniProt Accession	log_2_FC (Treated)	BH-FDR (Treated)	log_2_FC (Untreated)	BH-FDR (Untreated)	Category
IGHG4	P01861	−3.82	4.62 × 10^−13^	−2.92	6.62 × 10^−8^	Shared
C1R	B4DPQ0	2.42	4.62 × 10^−13^	2.71	3.61 × 10^−12^	Shared
C9	A0A8Q3SI95	2.22	6.98 × 10^−10^	1.78	3.74 × 10^−6^	Shared
IGLV1-47	P01700	−1.85	1.52 × 10^−8^	−2.06	5.09 × 10^−8^	Shared
CLU	P10909	1.67	3.02 × 10^−8^	1.54	5.19 × 10^−6^	Shared
IGKV3D-11	A0A0A0MRZ8	−1.27	1.17 × 10^−7^	−1.49	9.43 × 10^−8^	Shared
IGHG3	A0A9H3ZR93	−1.51	3.13 × 10^−6^	−2.70	2.28 × 10^−10^	Shared
HBA1; HBA2	P69905	1.66	3.64 × 10^−5^	2.10	1.14 × 10^−5^	Shared
FN1	P02751	2.38	9.54 × 10^−5^	2.71	1.74 × 10^−4^	Shared
C1S	P09871	1.03	1.34 × 10^−4^	1.16	2.67 × 10^−4^	Shared
PGLYRP2	Q96PD5	1.39	2.02 × 10^−4^	2.06	6.65 × 10^−6^	Shared
IGKV1-5	P01602	−2.14	2.96 × 10^−4^	−2.01	3.97 × 10^−3^	Shared
SERPINA1	P01009	−1.14	1.36 × 10^−3^	−1.65	1.00 × 10^−4^	Shared
KLKB1	P03952	−1.31	1.54 × 10^−8^	—	—	Only treated
C4BPA	P04003	1.10	7.15 × 10^−8^	—	—	Only treated
SERPINC1	P01008	1.09	2.56 × 10^−7^	—	—	Only treated
HPX	P02790	1.09	1.12 × 10^−6^	—	—	Only treated
IGLC2	P0DOY2	−1.30	1.14 × 10^−5^	—	—	Only treated
APOA2	V9GYM3	−1.03	1.35 × 10^−5^	—	—	Only treated
ORM2	P19652	1.24	2.53 × 10^−5^	—	—	Only treated
AMBP	P02760	−1.02	4.35 × 10^−5^	—	—	Only treated
IGHG2	P01859	1.59	3.63 × 10^−3^	—	—	Only treated
ALB	P02768	—	—	−1.13	2.61 × 10^−8^	Only untreated
APOA1	P02647	—	—	−1.03	6.88 × 10^−8^	Only untreated
IGHV3-30	P01768	—	—	−1.15	3.66 × 10^−5^	Only untreated
CPN2	P22792	—	—	1.46	3.08 × 10^−4^	Only untreated
IGHM	P01871	—	—	−1.28	2.48 × 10^−3^	Only untreated
LUM	P51884	—	—	1.47	4.92 × 10^−3^	Only untreated

## Data Availability

Dataset available on request from the authors.

## Supplementary Materials

The following supporting information can be downloaded at: https://www.mdpi.com/article/10.3390/biology15151271/s1, Figure S1: Distribution of plasma protein abundances before and after missing-value imputation; Figure S2: Individual plasma LFQ intensities of differentially abundant proteins in healthy controls and treated and untreated AKU patients; Table S1: Complete list of identified plasma proteins, identification parameters, peptide-support metrics, and participant-level abundance values; Table S2: Clinical, genetic, and biochemical characteristics and nitisinone treatment status of individual AKU patients.

## Abbreviations

The following abbreviations are used in this manuscript:

AA	Amyloid A
AKU	Alkaptonuria
BCA	Bicinchoninic acid
BH	Benjamini–Hochberg
BP	Biological Process
CC	Cellular Component
DDA	Data-dependent acquisition
DTT	Dithiothreitol
FDR	False discovery rate
FC	Fold change
GO	Gene Ontology
HGA	Homogentisic acid
HGD	Homogentisate 1,2-dioxygenase
IAA	Iodoacetamide
IgG	Immunoglobulin G
k-NN	k-nearest neighbour
LC-MS/MS	Liquid chromatography-tandem mass spectrometry
LFQ	Label-free quantification
MF	Molecular Function
MS	Mass spectrometry
MS/MS	Tandem mass spectrometry
PCA	Principal Component Analysis
SAA	Serum amyloid A
SPE	Solid-phase extraction
UHPLC	Ultra-high-performance liquid chromatography
