# Supplementary material for: Plasma Proteomic Signatures in Alkaptonuria
Source: Biology (Basel). 2026 Aug 3;15(15):1271. doi: 10.3390/biology15151271 (PMC13466101; doi:10.3390/biology15151271)
Supplement: Supplementary file 1 [file biology-15-01271-s001.zip › SI.pdf]

Supporting Information for

Plasma proteomic signatures in Alkaptonuria

Rebecca Finetti [1], Anna Visibelli [1,*], Bianca Roncaglia [1], Alfonso Trezza [1], Luana Peruzzi [1,2], Barbara Marzocchi[1,3], Ottavia Spiga [1,4] , Mattia Cicogni [5] , Laura Salvini [5], Laura Tinti [5], Vittoria Cicaloni [5], Sara Cheleschi [5], Marco Rossi [5], Cristina Tinti [5], and Annalisa Santucci [1,6,7]

[1]Department of Biotechnology, Chemistry and Pharmacy, University of Siena, 53100 Siena, Italy;
[2] UOC Medicina Molecolare e Genetica, Azienda Ospedaliera Senese, Siena, Italy;
[3] UOC Laboratorio Patologia Clinica, Ospedale S. Maria alle Scotte, AOU Senese, Siena, Italy;
[4] Centro della Scienza e della Tecnica, Polo Universitario Grossetano, Via Ginori 41, 58100 Grosseto, Italy;
[5] Fondazione Toscana Life Sciences, Strada del Petriccio e Belriguardo, 53100 Siena, Italy;
[6] Industry 4.0 Competence Center ARTES 4.0 Viale Rinaldo Piaggio, 56025 Pontedera, Italy;
[7] MetabERN, Department of Biotechnology, Chemistry and Pharmacy, University of Siena, Via Aldo Moro, 53100 Siena, Italy;
*Correspondence: anna.visibelli2@unisi.it

Table S1. See the attached .xlsx file “S1.xlsx”

Table S2. See the attached .xlsx file “S2.xlsx”

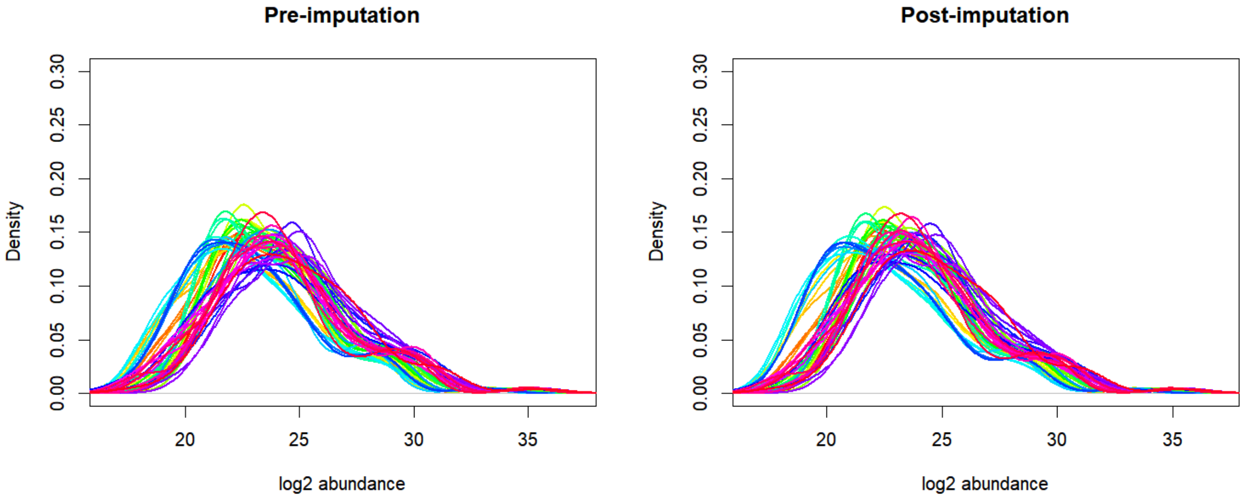

Figure S1. Distribution of plasma protein abundances before and after missing-value imputation. Density distributions of log_2-transformed protein abundances for individual samples are shown before imputation (left panel) and after k-NN imputation of missing values (right panel). Each curve represents one plasma sample.

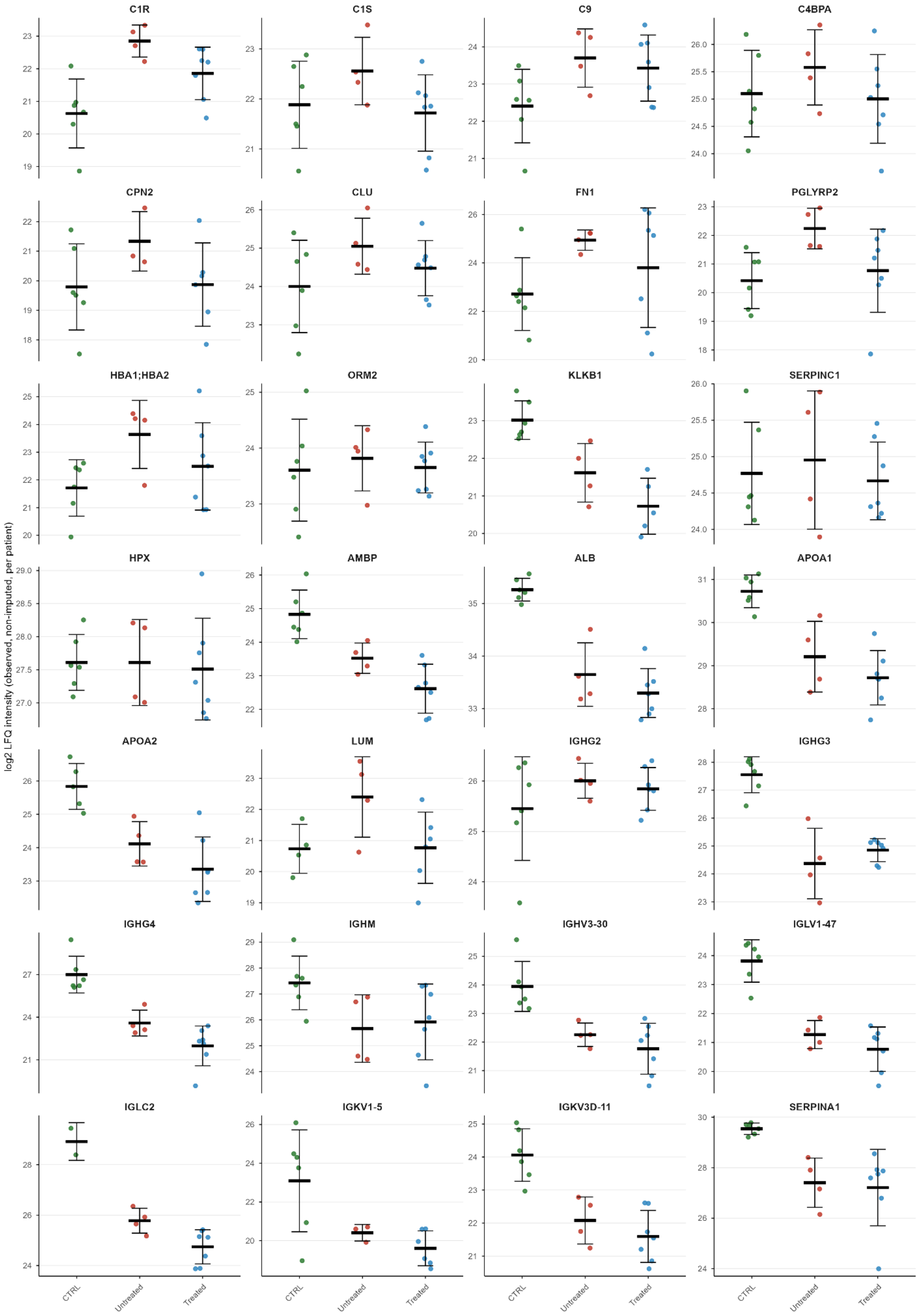

Figure S2. Individual plasma LFQ intensities (log2, observed values only, non-imputed) for the proteins differentially abundant between AKU patients and healthy controls (Table 2) and between treated/untreated

AKU patients and controls (Table 3). Each point represents one participant (mean of available technical replicates), black bars indicate mean ± SD calculated on observed values only. Green: healthy controls; red: untreated AKU patients; blue: treated AKU patients.
